# A comprehensive review of the relationship between autophagy and sorafenib-resistance in hepatocellular carcinoma: ferroptosis is noteworthy

**DOI:** 10.3389/fcell.2023.1156383

**Published:** 2023-04-27

**Authors:** Kangnan Zhang, Qinghui Zhang, Rongrong Jia, Shihao Xiang, Ling Xu

**Affiliations:** ^1^ Department of Gastroenterology, Tongren Hospital, Shanghai Jiao Tong University School of Medicine, Shanghai, China; ^2^ Department of Clinical Laboratory, Tongren Hospital, Shanghai Jiao Tong University School of Medicine, Shanghai, China

**Keywords:** hepatocellular carcinoma, autophagy, sorafenib-resistant, ferroptosis, cell

## Abstract

Patients with hepatocellular carcinoma (HCC) bear a heavy burden of disease and economic burden but have fewer treatment options. Sorafenib, a multi-kinase inhibitor, is the only approved drug that can be used to limit the progression of inoperable or distant metastatic HCC. However, enhanced autophagy and other molecular mechanisms after sorafenib exposure further induce drug resistance in HCC patients. Sorafenib-associated autophagy also generates a series of biomarkers, which may represent that autophagy is a critical section of sorafenib-resistance in HCC. Furthermore, many classic signaling pathways have been found to be involved in sorafenib-associated autophagy, including the HIF/mTOR signaling pathway, endoplasmic reticulum stress, and sphingolipid signaling, among others. In turn, autophagy also provokes autophagic activity in components of the tumor microenvironment, including tumor cells and stem cells, further impacting sorafenib-resistance in HCC through a special autophagic cell death process called ferroptosis. In this review, we summarized the latest research progress and molecular mechanisms of sorafenib-resistance-associated autophagy in detail, providing new insights and ideas for unraveling the dilemma of sorafenib-resistance in HCC.

## 1 Background

Hepatocellular carcinoma (HCC) has shown the fastest increasing mortality rate for decades (Siegel et al., 2022), with a high recurrence rate and low 5-year survival rate. While early HCC can be treated through tumor resection, liver transplantation, and other surgical treatments, more than 50% of HCC patients are diagnosed as advanced, and 70% of them relapse within the first 5 years of initial treatment (Forner et al., 2018). Advanced HCC requires a combination of local treatments (ablation, transcatheter arterial chemoembolization, external irradiation) and systemic treatment with sorafenib (Li and Wang, 2016). Although targeted therapy, systemic chemotherapy, and many other drugs have been used for the treatment of advanced liver cancer, they all have their own shortcomings to a greater or lesser extent. It is worth mentioning that lenvatinib is approved as a first-line systemic treatment for unresectable advanced liver cancer, as a recent clinical trial has shown that in untreated advanced HCC, the median survival time of lenvatinib for 13.6 months is no less than that of sorafenib (Zhao et al., 2020). Currently, lenvatinib is the only drug that is not inferior to sorafenib in the treatment of advanced liver cancer. However, compared to sorafenib, lenvatinib still has certain drawbacks. The median duration of treatment with lenvatinib is 1.5 times longer than that of sorafenib, which may increase the incidence of adverse events such as hypertension, proteinuria, dysphonia, and hypothyroidism (Kudo et al., 2018). Undoubtedly, lenvatinib is highly anticipated as a first-line systemic treatment for inoperable HCC patients. However, further clinical experience may be required to fully demonstrate whether lenvatinib can replace sorafenib (Al-Salama et al., 2019). Sorafenib is a small molecule multi-kinase inhibitor, sorafenib inhibits the proliferation of tumor cells by blocking the activities of Raf-1, B-Raf, and kinase in the Ras/Raf/MEK/ERK signaling pathway. In addition, sorafenib targets platelet-derived growth factor receptor (PDGFR-β), vascular endothelial growth factor receptor 2 and 3 (VEGFR-2, VEGFR-3), and hepatocyte factor receptor (c-kit) thereby diminishing tumorigenesis (Prieto-Dominguez et al., 2016). While in clinical studies, sorafenib was effective in prolonging median survival of patients with advanced HCC (Abdelgalil et al., 2019), the resistance response of patients to sorafenib further limits the drug’s efficacy. Sorafenib-resistance has become a major obstacle in the clinical treatment of advanced HCC patients, making it particularly important to understand the mechanism of sorafenib-resistance in HCC.

**FIGURE 1:**
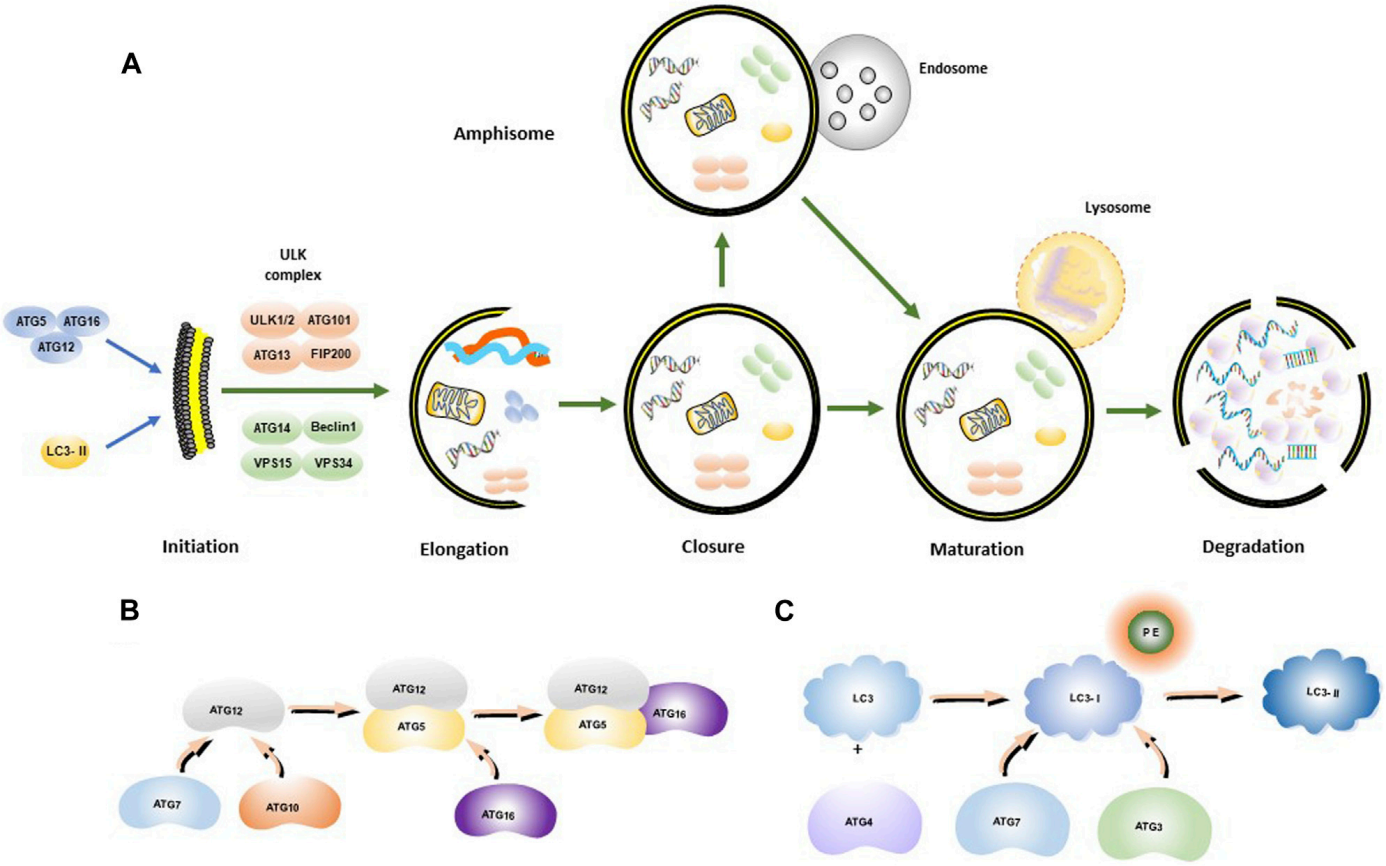
Autophagosome synthesis and autophagy process. **(A)** Schematic model of autophagic flux; **(B)** ATG12-ATG5-ATG16 complex formation process; **(C)** LC3-II formation process.

Autophagy is a cellular degradation and recycling process that is highly conserved in all eukaryotes. Researchers have found that cancer cells may mobilize this procedure for drug resistance to sorafenib (Kwanten et al., 2014). In mammalian cells, there are three main types of autophagy: microautophagy, macroautophagy, and chaperone-mediated autophagy (CMA) (Zhong et al., 2009; Yang and Klionsky, 2010a). Autophagy often induced by various stresses in the human body as a self-protective mechanism, which is involved in cell homeostasis and cell composition (Huang et al., 2018) Through promoting the removal or renewal of long living or misfolded proteins, protein aggregates and damaged organelles, the key processes of autophagy are broadly divided into five steps (Figure 1A), including initiation, elongation, closure, maturation, and degradation, and finally the release of degradation products back into the cytoplasm (He and Klionsky, 2009; Yang and Klionsky, 2010b). Autophagy is initiated by the separation of membranes or nucleation of phagosomes, which is triggered by induction of the ULK1-ATG13-FIP200 complex, composed of ATGs (Figure 1B) and IL3-II (Figure 1C). The phagosome is then elongated with the help of the ULK complex and the class III PtdIns3K complex (Beclin1-Vps34-ATG14). The class III PtdIns3K complex facilitates the formation and elongation of the separation membrane. Elongation of the phagocyte membranes is dependent on the ATG5-ATG12-ATG16 and LC3 binding systems. The separation membrane is then blocked by LC3-II to form autophagosomes. The outer membrane of the autophagosome fuses with the lysosomal membrane, and in some cases, the autophagosome may fuse with the endosome to form an amphisome, which, in turn, fuses with a lysosome to form an autolysosome. The contents of the autolysosome are finally degraded by lysosomal hydrolases.

Autophagy progression can be promoted or inhibited in different cancers, indicating that autophagy is a manipulable program that can affect cancer cell survival (Yazdani et al., 2019). However, whether autophagy acts as an anti-cancer or a tumor-promoting mechanism remains controversial (Cui et al., 2013). In this review, we elaborate on the mechanism of sorafenib-induced autophagy, changes in intercellular communication following sorafenib exposure, and how autophagy remodels the HCC tumor microenvironment. Intriguingly, we suggest that ferroptosis is also a neglected form of autophagy during sorafenib resistance. The relevant signaling pathways and biomarkers in the process of autophagy may further serve as potential targets to reverse sorafenib resistance and predict HCC prognosis.

## 2 The dual role of autophagy in HCC

**FIGURE 2:**
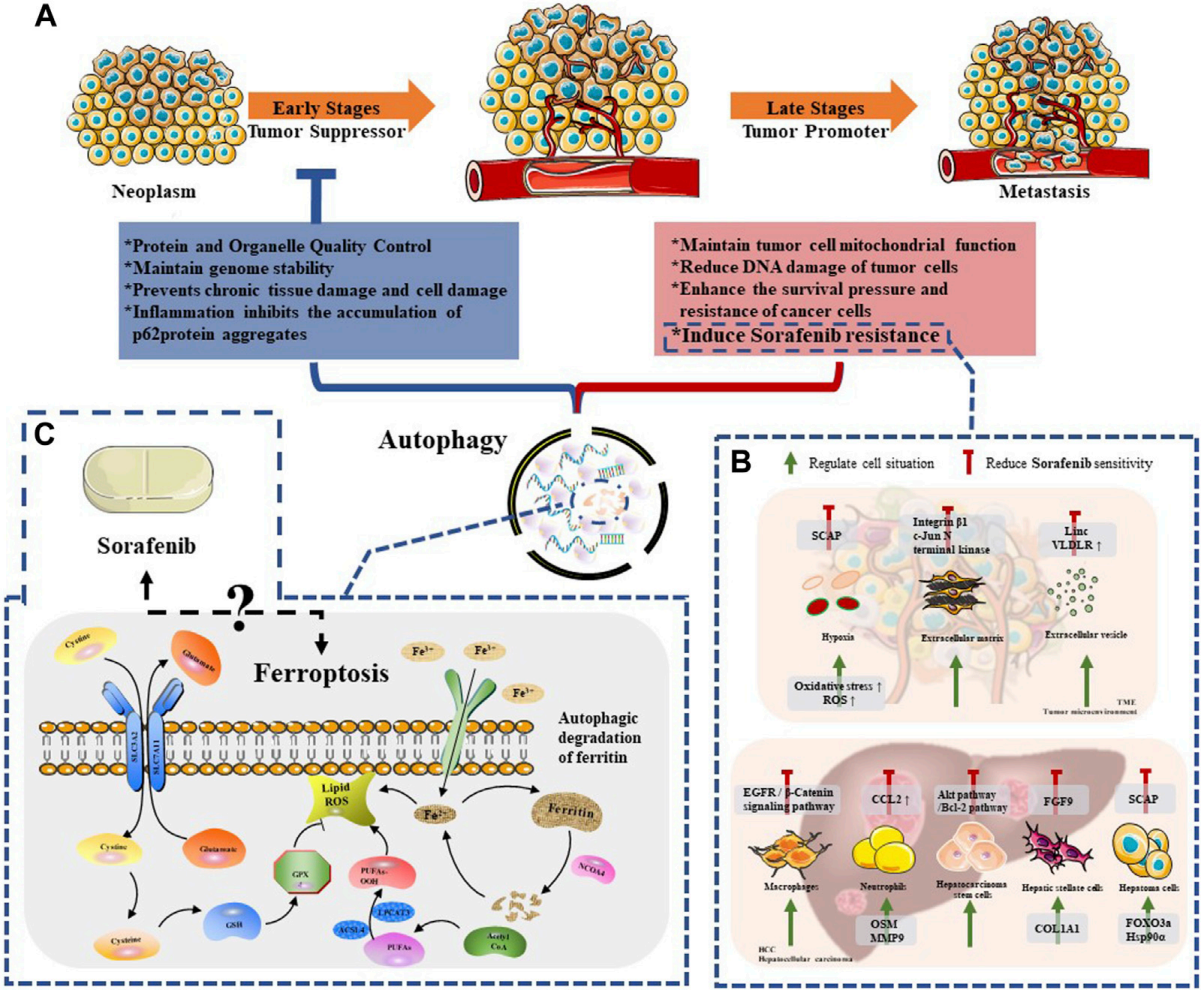
The relationship between autophagy and Sorafenib therapy. **(A)** The dule role of autophagy in neoplasm progression; **(B)** The regulation mechanisms of autophagy among HCC cells and TME compartments, which reduced Sorafenib sensitivity; **(C)** The mechanism of ferroptosis and the remaining issues between this special mode of autophagy and sorafenib.

Since the study of yeast identified the core autophagy-related proteins, the molecular era of autophagy research has begun (Titorenko et al., 1995). Dysregulated autophagy has been found to be related to various diseases, including neurodegenerative diseases (Chu, 2019), cardiovascular diseases (Abdellatif et al., 2020), gastrointestinal diseases (Cao et al., 2019), lung diseases (Rezaei et al., 2020), cancer (Li et al., 2020) and other diseases. Therefore, a more thorough understanding of autophagy is better for the treatment of these diseases. With the deepening of research, it has been found that the process of autophagy is extremely complex (Figure 2A). In the occurrence and development of cancer, many studies indicated that autophagy plays a dual role (Li et al., 2020), and whether autophagy acts as an anti-tumor or a pro-tumor mechanism remains controversial (Cui et al., 2013). In the early stages of tumorigenesis (Barnard et al., 2016), autophagy functions as a protein and organelle quality control system that maintains genomic stability, protects against chronic tissue damage, and inhibits inflammation-related accumulation of oncogenic p62 protein, thereby preventing tumor initiation, proliferation, invasion, and metastasis (Guo et al., 2013). Studies have demonstrated that artificially inhibiting autophagy progression (through ATG5 knockout) would enhance the growth of tumors at an early stage in the liver, indicating that autophagy suppression in hepatocytes relies on tumor suppression (Takamura et al., 2011). However, once a tumor develops into an advanced stage, autophagy turns into a protector of tumor cells, reducing DNA damage and improving cancer cell survival by inducing drug resistance (White, 2012; Wu et al., 2012). For instance, Liu et al. found that autophagy inhibits TP53 and induces the expression of the transcription factor NANOG to immortalize hepatoma stem cells and promote hepatocarcinogenesis in benign liver tumors (Liu et al., 2018). Therefore, we believe that autophagy is a weapon that we can manipulate to curb early tumor progression and reverse late tumor drug resistance. However, further exploration is required to identify precise methods to regulate autophagy effectively as a treatment strategy for HCC.

## 3 Autophagy gradually controls the whole field during sorafenib-resistance in HCC

Sorafenib is a novel molecular targeted therapy drug, which can target tumor cells and tumor vascular receptors. Therefore, sorafenib can significantly inhibit tumor cell proliferation on the one hand; on the other hand, it can significantly inhibit tumor angiogenesis. In other words, it can “kill two birds with one stone” and play the dual role of anti-angiogenesis and anti-tumor cell proliferation at the same time (Prieto-Dominguez et al., 2016). Despite its effectiveness, the clinical efficacy of sorafenib is mainly limited by the development of drug resistance. The mechanisms of resistance include metabolic reprogramming, dysregulation of PI3K/AKT and JAK/STAT pathways, epithelial-mesenchymal transition, and hypoxia-induced responses caused by sorafenib’s inhibition of angiogenesis. Furthermore, recent studies have shown that autophagy-induced resistance may be a new way for hepatoma cells to resist sorafenib (Figure 2B). Therefore, it is essential to clarify the relationship between sorafenib-induced autophagy and drug resistance in the treatment of patients with advanced HCC.

### 3.1 Efficacy of sorafenib in combination with other drugs in the treatment of liver cancer

The complex molecular pathogenesis of HCC has also led researchers to shift their focus towards the combination therapy of sorafenib. Sorafenib has been combined with MEK/ERK pathway inhibitors, anti-angiogenic agents, mTOR pathway inhibitors, histone deacetylase inhibitors, HGF/c-Met pathway inhibitors, and EGF/EGFR pathway inhibitors (Gao et al., 2015; Huang et al., 2020). Other drugs, such as interferon (Itokawa et al., 2016), capecitabine (Patt et al., 2017), gemcitabine and oxaliplatin (GEMOX) (Liu et al., 2015; Assenat et al., 2019) have been studied. However, to date, none of the combinations have achieved satisfactory results in the third phase of clinical trials. Given that sorafenib and transcatheter arterial chemoembolization (TACE) are recommended therapies for advanced liver cancer, researchers have proposed that combining them may result in better treatment outcomes than either therapy alone. A Chinese study showed that the combination of sorafenib and TACE increased overall survival by more than 50% compared to TACE alone (Qu et al., 2012). This finding is supported by other investigations (Varghese et al., 2017; Ren et al., 2019; Yuan et al., 2019). However, results vary based on regional differences and heterogeneity of trial protocols. In a multicenter, randomized, placebo-controlled phase III European trial, adding sorafenib treatment did not improve progression-free survival (PFS) compared to TACE alone (Lencioni et al., 2016; Meyer et al., 2017). There is even evidence to suggest that sorafenib provides no survival benefit for unresectable HCC patients undergoing TACE (Kudo et al., 2011).

Therefore, finding a solution to sorafenib resistance in the treatment of liver cancer is critical. Currently, there is no solid evidence to suggest that combining sorafenib with other drugs can solve the problem of resistance or even improve its efficacy.

### 3.2 Sorafenib induces autophagy in HCC

To address the issue of sorafenib resistance in advanced liver cancer patients, it is essential to understand the reasons behind it. Even gaining a small insight into the problem can be significant in overcoming resistance. One important aspect to consider is the relationship between sorafenib and autophagy during the treatment process. While the potential mechanism of sorafenib-induced autophagy is not fully understood, some signaling pathways have been identified (Table 1). The mTOR pathway is one of the primary regulators of cell metabolism in response to oxidative stress, nutrient deficiency, and growth factor deficiency (Neufeld, 2010; Yang and Ming, 2012). Studies have shown that sorafenib can inhibit the mTORC1 pathway, leading to the induction of autophagy (Shimizu et al., 2012). Another study revealed that sorafenib induced apoptosis and autophagy of human hepatoma cells through endoplasmic reticulum (ER) stress. The upregulation of IRE1 signal induced by sorafenib was important for the induction of autophagy, while both ER stress and autophagy were related to cell death induced by sorafenib in hepatoma cells (Shi et al., 2011). Beclin-1 is one of the key proteins of autophagy progress (Yuan et al., 2013) Research has shown that sorafenib and its derivative sc-59 induce hepatoma autophagy through the SHP-1/STAT3/MCL-1/Beclin-1 pathway. In multi-HCC cell lines, sorafenib downregulates phosphorylated STAT3 (p-STAT3) and decreases the expression of myeloid leukemia-1 (MCL-1), thereby breaking down the Beclin1-MCL-1 complex and inducing autophagy. Meanwhile, sc-59 also downregulates p-STAT3 and induces autophagy (Tai et al., 2013). Recent studies have also shown that autophagy may help inhibit cell proliferation treated with sorafenib by affecting the HIF/mTOR signaling pathway. Yang et al. found that both sorafenib and hypoxia induce cell autophagy through the HIF/mTOR-related signaling pathway (Yang et al., 2021). Additionally, sorafenib can hijack sphingolipids, the bioactive lipids that are involved in many cellular pathways such as apoptosis, cell cycle, aging, or cell differentiation regulation (Hannun and Obeid, 2018), to induce autophagy. The level of sphingosine-1-phosphate (S1P) in mice with HepG2 xenograft tumors treated with sorafenib decreased slightly, which promotes the fusion of lysosomes and autophagosomes (Beljanski et al., 2011; Harvald et al., 2015). Therefore, sorafenib has some effects on the imbalance of sphingolipid metabolism, which will affect the occurrence of autophagy. Furthermore, sorafenib may induce autophagy by affecting the transcription of microRNA (miRNA) (Miska, 2005). MiRNA-423-5p plays a role in cell cycle regulation and autophagy in HCC cells. Paola et al. found that the level of miRNA-423-5p in the serum of HCC patients increased after sorafenib treatment (Stiuso et al., 2015). This suggests that sorafenib could upregulate the transcription of miRNA-423-5p, leading to the induction of autophagy.

**TABLE 1:** Sorafenib induces autophagy in HCC.

Influence factor	Cell line	Signal pathway	Result	References
mTOR Pathway	Huh7, HLF, PLC/PRF/5	PI3K/Akt pathway and mTOR pathway	Sorafenib can not only induce autophagosome formation, but also activate autophagy flux	Liu et al. (2018)
	HepG2	HIF-1/mTOR related signal pathway	Sorafenib can inhibit the proliferation of hepatoma cells and induce autophagy and apoptosis of hepatoma cells through HIF-1/mTOR related signal pathway	Patt et al. (2017)
Endoplasmic Reticulum Stress	MHCC97-L, PLC/PRF/5, HepG2	Sorafenib induced ER stress and upregulated IRE1 signal	Sorafenib induces apoptosis and autophagy of human hepatoma cells by causing endoplasmic reticulum (ER) stress, which is independent of MEK1/2-ERK1/2 pathway	Huang et al. (2020)
Beclin-1Protein	PLC5, Sk-Hep1, HepG2, Hep3B	SHP-1-STAT3-Mcl-1-Beclin1 pathway	Sorafenib and its derivatives induce the inhibition of ml-1 through SHP-1/STAT3 related pathway and release Beclin1 to promote the formation of autophagosome	Itokawa et al. (2016)
miR-423-5p	Huh7, HepG2	—	Sorafenib can induce autophagy by affecting mir-423-5p in the treatment of hepatocellular carcinoma	Ren et al. (2019)
Sphingosine-1-phosphate (S1P)	HepG2 SK-HEP1, Hep 3b2.1-7	Cell proliferation pathways (such as MAP/ERK pathway)	Sorafenib has some effects on the imbalance of sphingolipid metabolism, which will affect the occurrence of autophagy	Qu et al. (2012)

### 3.3 Autophagy in turn regulates cell-crosstalk to promote sorafenib-resistance in HCC

It is well known that autophagy plays a dual role in cancer progression. In hepatocellular carcinoma (HCC), high autophagy flux induced by sorafenib treatment can result in the upregulation of autophagy-related proteins, which may subtly affect cell-to-cell communication and the entire tumor microenvironment (Liu et al., 2011; Parkhitko et al., 2013).

However, autophagy triggered by liver cancer cells resistant to sorafenib can lead to further drug resistance. For example, He et al. discovered that sorafenib-resistant hepatoma cells can upregulate miR-21, which in turn inhibits autophagy induced by sorafenib through downregulation of PTEN and altering the activation sequence of Akt pathway. This leads to drug resistance, as confirmed by transfecting miR-21 mimics into parent HCC cells, making them insensitive to sorafenib by inhibiting autophagy (He et al., 2015). Similarly, Chen et al. found that colorectal neoplasia differentially expressed (CRNDE) plays a critical role in regulating autophagy and drug resistance to sorafenib in hepatoma cells. Sorafenib activates the CRNDE/ATG4B/autophagy pathway, and inhibiting CRNDE reduces autophagy occurrence, making HCC cells sensitive to sorafenib. Targeting the CRNDE/ATG4B/autophagy pathway may be a promising strategy to improve sorafenib sensitivity in HCC (Chen et al., 2021). Transcription factors are also involved in regulating the sorafenib-autophagy and resistance process. Knocking out FOXO3a in HCC xenograft tumors significantly improves the efficacy of sorafenib by inhibiting autophagy. Yan et al. found that Hsp90α plays a key role in sorafenib-resistance by down-regulating HSP90α, which binds to the necrotic complex, promoting chaperone-mediated autophagy and increasing sorafenib resistance (Liao et al., 2021). Recent studies by Li et al. have demonstrated that the cholesterol sensor SCAP can participate in drug resistance to sorafenib through AMPK-mediated autophagy regulation. Inhibition of SCAP improves the sensitivity of sorafenib-resistant HCC cells, as SCAP is overexpressed in sorafenib-resistant HCC tissues and hepatoma cell lines (Li et al., 2022). Clearly, following resistance to sorafenib in HCC, there appears to be a series of positive feedback from autophagy that leads to continued spread of sorafenib resistance.

Liver cancer stem cells (LCSCs) have the ability to self-renew and differentiate, which is involved in tumor progression by regulating stemness, drug resistance, and angiogenesis (Wong et al., 2021). In HCC, the CD133+ subtype of CSC cells has been characterized as being involved in the activation of Akt molecular pathway and the b-lymphoma-2 (Bcl-2) cell survival pathway to resist cytotoxicity from chemotherapy drugs such as sorafenib (Ma et al., 2008). Autophagy is involved in maintaining CD133+ LCSCs under hypoxic and nutrient conditions, resulting in sorafenib resistance during HCC treatment (Song et al., 2013).

Studies have shown that hepatic stellate cells (HSCs) activation is closely related to the increase of autophagy (Thoen et al., 2011), which is a critical step in the process of HSC activation (Lucantoni et al., 2021). FGF9 extracted from activated HSCs has been found to enhance the tumorigenicity and drug resistance of HCC cells (Seitz et al., 2020). At the same time, Song et al. found that the interaction between HCC cells and HSCs promoted the firmness of HCC microenvironment by accumulating collagen 1A1 (COL1A1), also resulting in the drug resistance of HCC. Moreover, the interaction between HCC cells and HSCs can promote the firmness of HCC microenvironment by accumulating collagen 1A1 (COL1A1), which also results in drug resistance of HCC. Although the mechanisms involved in HSC-related autophagy remain to be explored, the activation of HSCs may increase the promotion of sorafenib-resistance during HCC treatment (Song et al., 2016).

Besides, compared with normal endothelium, the sensitivity of endothelial cells of tumor vessels to some chemotherapeutic drugs is reduced (Bani et al., 2017). Tumor-derived endothelial cells (TEC) with unique phenotypes in HCC have been found to obtain drug resistance during sorafenib treatment (Xiong et al., 2009). Therefore, regulating the effect of autophagy on tumor vascular endothelial cells is of great significance to alleviate sorafenib-resistance.

Autophagy can also lead to sorafenib-resistance in liver cancer by affecting immune cells. Macrophage autophagy has been found to be beneficial to hepatocarcinogenesis by inducing immunosuppressive microenvironment (Deust et al., 2021). Tumor-associated macrophages have been found to regulate epidermal growth factor receptor (EGFR)/β-Catenin signaling pathway to promote cell proliferation, invasion and sorafenib-resistance (Wei et al., 2017). These results indicated that macrophage autophagy could create better condition for hepatocarcinogenesis, and macrophage can promote sorafenib-resistance under specific conditions. In addition, neutrophil autophagy also has tumor-promoting and anti-tumor functions, depending on the tumor environment (Yu and Sun, 2020). For example, neutrophil autophagy promoted HCC tumor growth and migration by increasing the levels of pro-metastatic proteins Oncostatin M (OSM) and MMP-9 (Li et al., 2015). Neutrophils have been found to recruit macrophages and Tregs cells to HCC by secreting C-C motif chemokine ligand 2 (CCL2) and C-C motif chemokine ligand 17 (CCL17), promoting neovascularization, growth, metastasis, and resistance to sorafenib (Zhou et al., 2016). Thus, further exploration is needed to determine whether neutrophil autophagy affects sorafenib-resistance.

As described above, autophagy interacts with various cells involved in the cancer progression, impacting the effectiveness of sorafenib. Mobilizing the crosstalk between autophagy and various cells in liver cancer could provide new insights for the treatment of HCC.

### 3.4 Autophagy rebuilding the microenvironment to promote sorafenib-resistance

Tumor microenvironment (TME) has become a focal point in cancer research and drug development due to its pivotal role in cancer development and treatment (Xiao and Yu, 2021). Autophagy, which exploits the plasticity of TME, is known to promote resistance of hepatoma cells to sorafenib (Sun et al., 2017; Tang et al., 2020). In addition to its crosstalk with immune cells, autophagy also regulates hypoxia, extracellular matrix, and extracellular vesicles in TME to promote drug resistance.

Hypoxia in TME requires the regulation of hypoxia-inducible factor (HIF) to adapt to the hypoxic environment (Ju et al., 2016). Mitophagy, a specialized form of autophagy, is activated under hypoxic conditions in HCC cells through the regulation of HIF-1α, which contributes to sorafenib-resistance and protects tumor cells (Prieto-Domínguez et al., 2017). Specifically, researchers have shown that the β-2 adrenergic receptor (ADRB2) signaling is disrupted in an Akt-dependent manner with the help of beclin1/phosphatidylinositol-3-kinase VPS3/autophagy-related protein 14 complex, which stabilizes HIF-1α and leads to sorafenib-resistance (Wu et al., 2016). Therefore, regulating HIF with autophagy as a starting point and then changing the hypoxic state of TME and alleviating tumor resistance may be feasible reversal strategies.

Extracellular matrix (ECM) is a dynamic network composed of biophysical and biochemical factors that maintain tissue homeostasis (Karamanos et al., 2019). Recent research has identified several ECM-derived proteoglycans and proteins as strong inducers of autophagy (Karamanos et al., 2019), while autophagy itself affects ECM function (Chen and Iozzo, 2022). Studies by Nguyen et al. have shown that a collagen-rich tumor microenvironment (TME) is involved in sorafenib resistance during tumor sclerosis through integrin β1 and its downstream effector, c-Jun N-terminal kinase (Nguyen et al., 2014). Similarly, laminin-332 (Ln-332) produced by HSC acts as a ligand for α3β1 and α6β4 integrins on the surface of HCC cells, causing ubiquitination of focal adhesion kinase (FAK) and promoting sorafenib-resistance (Azzariti et al., 2016). In addition, extracellular vesicles also contribute to sorafenib resistance, and intracellular autophagy plays a role in determining the contents and release process of these vesicles under various stimuli (Zheng et al., 2019). Hepatoma cell-derived microvesicles (MVs) can induce sorafenib-resistance *in vitro and in vivo* (Jaffar Ali et al., 2021). Takahashi et al. identified lincRNA-VLDLR as an extracellular vesicle-rich lncRNA that contributes to cellular stress responses. They also found that lincRNA-VLDLR was significantly upregulated in EVs. When HCC cells were exposed to the anticancer agent such ad sorafenib (Takahashi et al., 2014).

Autophagy exerts significant impacts on sorafenib efficacy during HCC treatment, not only intracellularly but also in TME, which mobilizes all available materials in tumors and adjacent non-cancerous areas to hinder sorafenib and control the whole field to promote advanced HCC progression. Therefore, identifying effective mechanisms or targetable signaling pathways that regulate autophagy and thereby reverse sorafenib-resistance is currently an important topic that urgently needs to be addressed.

## 4 Novel autophagic mechanisms induce sorafenib-resistance in HCC

### 4.1 The autophagic cell death process: ferroptosis

Ferroptosis is a type of regulated necrosis that has been discovered in recent years. Unlike apoptosis or necrosis, it does not depend on caspase activity or receptor-interacting protein 1 (RIPK1) kinase activity (Bebber et al., 2020). The hallmark morphological features of ferroptosis include cell contraction and increased mitochondrial membrane density (Fricker et al., 2018). While the precise mechanism of ferroptosis is not fully understood, studies have shown that the regulation of ferroptosis is mainly mediated by cystine glutamate reverse transporter (System X_C_^-^) and glutathione peroxidase 4 (GPx4) (Figure 2C). System X_C_-is a heterodimeric cystine/glutamate antiporter composed of two core components: SLC7A11 (solute carrier family 7 members 11; catalytic subunit) and SLC3A2 (solute carrier family 3 member 2; anchor protein). This amino acid antiporter maintains the intracellular redox state by importing cystine, which is then reduced to cysteine and used to synthesize the major antioxidant glutathione (GSH) (Song et al., 2018). GPx4 is a protein enzyme that inhibits lipid peroxidation and prevents ferroptosis by inhibiting the accumulation of lipid peroxides in cells. When GPx4 is inhibited, it can lead to the accumulation of reactive oxygen species (ROS) in cells and induce ferroptosis (Zou et al., 2019).

At the same time, some studies have found that autophagy can induce the occurrence of ferroptosis. Many studies have shown that autophagy is closely related to ferroptosis: Shen et al. revealed that YTHDF1 (m6A reader) triggers autophagy activation by identifying m6A binding sites to promote stability of becn1 mRNA, which eventually leads to Hepatic stellate cells (HSCs) ferroptosis (Shen et al., 2021). Similarly, Mou et al. found that autophagy can promote ferroptosis by producing lysosomal ROS (Mou et al., 2019). Some studies suggest that ferroptosis is a form of autophagic cell death (Torii et al., 2016; Kang and Tang, 2017). Gao et al. tested whether iron-related ROS production requires autophagy, especially lipid ROS accumulation. They found that both pharmacological and genetic inhibition of autophagy significantly inhibited the accumulation of lipid ROS associated with ferroptosis (Gao et al., 2016). They also found that NCOA4 (nuclear receptor coactivator 4) (Mancias et al., 2014) is a selective cargo receptor for the selective autophagic turnover of ferritin (namely, ferritinophagy) in ferroptosis. Knockout of NCOA4 or ATG (such as ATG3, ATG5, ATG7, and ATG13) inhibited rubber-induced ferritin degradation, iron accumulation and lipid peroxidation, and subsequent iron ptosis (Gao et al., 2016; Hou et al., 2016). Similarly, Hou et al. demonstrated that autophagy promotes ferroptosis through the degradation of ferritin in fibroblasts and cancer cells. They found that overexpression of NCOA4 increased the degradation of ferritin and promoted ferroptosis (Hou et al., 2016). Autophagy supplies available labile iron via NCOA4-mediated ferritinophagy to the process of ferroptosis (Lu et al., 2017). These findings demonstrate that ferroptosis is a selective autophagic cell death process.

### 4.2 Ferroptosis and sorafenib in HCC

It has been stated previously that ferroptosis is a form of selective autophagic cell death. However, research has also shown that ferroptosis is closely linked to sorafenib-resistance. Several studies have demonstrated that ferroptosis can synergize with sorafenib to kill hepatocellular carcinoma cells. Sorafenib induces mitochondrial dysfunction and oxidative stress, which may be associated with the activation of ferroptosis. Depletion of glutathione (GSH) caused by cysteine deprivation or cysteinase inhibition exacerbates sorafenib-induced ferroptosis and lipid peroxide production, leading to enhanced oxidative stress and mitochondrial ROS accumulation. Cysteine depletion may thus play a synergistic role with sorafenib by inducing iron-mediated ferroptosis (Li et al., 2021a). Li et al. have shown that artesunate and low-dose sorafenib induce ferroptosis and kill hepatocellular carcinoma cells by inducing oxidative stress and lysosomal activation *in vitro and in vivo.* This suggests that artesunate and sorafenib have a synergistic effect, providing a new possibility for overcoming drug resistance in hepatocellular carcinoma cells (Li et al., 2021b). Recent studies have found that sorafenib-induced mitochondrial dysfunction activates the PI3K-RAC1-PAK1 signal transduction pathway, leading to macrocytosis in human hepatocellular carcinoma specimens and xenograft tissues. Macrocytosis prevents sorafenib-induced ferroptosis by supplementing sorafenib to treat depleted intracellular cysteine, making hepatocellular carcinoma cells resistant to sorafenib. Finally, they used amiloride to inhibit large cell proliferation, which significantly sensitized drug-resistant tumors to sorafenib (Byun et al., 2022). These studies show that ferroptosis plays a critical role in the killing effect of sorafenib on hepatocellular carcinoma cells. By combining drugs that can promote the ferroptosis-inducing effect of sorafenib, the killing effect of sorafenib on hepatocellular carcinoma cells can be enhanced, providing a new approach for overcoming sorafenib drug resistance.

Several substances in the ferroptosis pathway can also be used to predict the prognosis of sorafenib treatment. Metallothionein (MT) is a small intracellular protein rich in cysteine that is widely expressed in eukaryotic cells. MT plays a critical role in heavy metal detoxification and antioxidation. Among them, MT1 is actively involved in resisting oxidative stress of various types of cells (Krizkova et al., 2018). Studies have found that MT1G can inhibit ferroptosis by resisting intracellular GSH depletion, resulting in sorafenib resistance in HCC patients. HCC patients with high MT1G expression have a poor prognosis after sorafenib treatment (Sun et al., 2016). Therefore, MT1G can be used as a potential prognostic index for patients with advanced HCC after sorafenib treatment. Li et al. identified CDGSH iron sulfur domain 2 (CISD2) as a new biomarker resistant to sorafenib-induced ferroptosis for the first time. CISD2 is highly expressed and is associated with sorafenib-resistance. CISD2 promotes resistance to sorafenib-induced ferroptosis by regulating Beclin1 in hepatoma cells (Li et al., 2021c). Their research provides valuable insights into the targeted treatment of sorafenib-resistant hepatocellular carcinoma (HCC) and ferroptosis. Specifically, the study shows that CISD2 is highly expressed in HCC patients and is associated with sorafenib resistance and poor prognosis. Another important finding is the identification of ABCC5 as a key regulator and a promising therapeutic target in acquired sorafenib resistance in human HCC cells. Huang et al. demonstrated that sorafenib upregulates ABCC5 through the PI3K/Akt/Nrf2 pathway, which inhibits lipid peroxidation-mediated ferroptosis and promotes cancer progression, leading to acquired sorafenib resistance in human HCC cells. Therefore, regulating ABCC5 expression to induce ferroptosis is a potential therapeutic strategy for overcoming acquired sorafenib resistance in hepatoma cells (Huang et al., 2021). Overall, these results suggest new avenues for improving sorafenib resistance and identifying prognostic markers in patients with HCC.

## 5 Conclusion

Liver cancer is one of the three leading causes of cancer death in 46 countries and one of the five leading causes of cancer death in 90 countries. In 2020, the population of East Asia will represent 21.5% of the global population, but half of the world’s liver cancer deaths will occur in East Asia (54.3% and 54.1% respectively). China alone has 45.3% of the world’s liver cancer cases and 47.1% of liver cancer deaths (Rumgay et al., 2022). Sorafenib has unique advantages in the treatment of advanced HCC, but due to its drug resistance, the curative effect on most patients is not as good as expected. This paper reviews the interaction between sorafenib and autophagy in HCC and discusses how autophagy affects HCC cells themselves, HCC stem cells, other parenchymal cells in HCC, tumor microenvironment, and autophagic cell death process: Ferroptosis, which leads to the possible mechanism and prognostic marker protein of sorafenib-resistance.

Numerous studies have demonstrated that autophagy plays a dual role in cancer, and that it can contribute to sorafenib resistance in multiple ways. Additionally, autophagy-induced ferroptosis has been shown to play a crucial role in reversing sorafenib resistance in hepatocellular carcinoma (HCC). While most research has indicated that sorafenib can enhance the lethality of liver cancer cells through ferroptosis, some studies have found that sorafenib does not trigger ferroptosis by inhibiting the system X_C_^−^ or by other mechanisms related to ferroptosis. To investigate whether sorafenib can function as a potent system X_C_^−^ inhibitor or ferroptosis inducer in various cell lines, Zheng et al. conducted an experimental study on the SLC7A11 gene in two cell lines: the human fibrosarcoma cell line HT1080, widely used in ferroptosis research, and the human embryonic kidney cell line HEK293T. Their findings showed that sorafenib did not induce ferroptosis in either cell line. Furthermore, their research indicated that sorafenib failed to trigger ferroptosis in cell lines with high expression of the cystine/glutamate antiporter (xCT). Given that sorafenib is the first-line drug for patients with advanced liver cancer, Zheng et al. investigated whether sorafenib causes ferroptosis in four human hepatoma cell lines (HLE, HLF, HepG2, and Huh7). Surprisingly, their experimental results showed that sorafenib did not induce ferroptosis in any of these cell lines. Therefore, the authors concluded that sorafenib is not a true ferroptosis inducer. Although the substrate-specific subunit SLC7A11 of the system X_C_^−^ is expressed significantly, ferroptosis induced by the system X_C_^−^ inhibitor can only be achieved in a small number of tumor cell lines (Zheng et al., 2021).

There are still many unanswered questions about the relationship between sorafenib and ferroptosis (as shown in Figure 2C). As mentioned earlier, existing research indicates that ferroptosis is a reliable induction pathway. It can be triggered by depleting cysteine (such as using amiloride to inhibit erythropoietin-induced erythrocyte proliferation, thereby preventing cysteine supplementation), using artemisinin, inhibiting MT1G, inhibiting CISD2, inhibiting ABCC5, and other methods to induce ferroptosis. This leads to the occurrence of new cell death in HCC that was previously resistant to apoptosis, thereby restraining tumor occurrence and development. However further research is needed to uncover the details of this relationship. Additionally, understanding the balance between ferroptosis and autophagy is crucial for overcoming drug resistance to sorafenib, making it an important area of study. However, while both ferroptosis and autophagy show potential, their roles in HCC cells themselves, HCC stem cells, other HCC parenchyma cells, and the tumor microenvironment have not been fully explored, including their targeted-potential mechanisms and related signaling pathways.It is worth noting that cuproptosis, a new mode of cell death, has recently been reported to be related to the development of ferroptosis. By disrupting the homeostasis of cuproptosis in HCC, it can inhibit HIF1a/CP ring to enhance ferroptosis. As a result, the relationship between cuproptosis and autophagy and the potential impact on drug resistance of sorafenib in liver cancer is also worth further exploration.

## Figures and Tables

**Table:** 

**HCC**	hepatocellular carcinoma
**PDGFR-β**	platelet-derived growth factor receptor
**VEGFR-2**	vascular endothelial growth factor receptor 2
**VEGFR-3**	vascular endothelial growth factor receptor 3
**CMA**	chaperone-mediated autophagy
**ER**	endoplasmic reticulum
**p-STAT3**	phosphorus STAT3
**MCL-1**	myeloid leukemia-1
**miRNA**	microRNA
**S1P**	sphingosine-1-phosphate
**LCSCs**	liver cancer stem cells
**Bcl-2**	b-lymphoma-2
**COL1A1**	collagen 1A1
**TEC**	tumor-derived endothelial cells
**EGFR**	epidermal growth factor receptor
**OSM**	Oncostatin M
**CCL2**	C-C motif chemokine ligand 2
**CCL17**	C-C motif chemokine ligand 17
**TME**	tumor microenvironment
**HIF**	hypoxia-inducible factor
**ADRB2**	β-2 adrenergic receptor
**ECM**	extracellular matrix
**Ln-332**	laminin-332
**FAK**	focal adhesion kinase
**MVs**	microvesicles
**ripk1**	receptor-interacting protein 1
**GPx4)**	glutathione peroxidase 4
**SLC7A11**	solute carrier family 7 members 11; catalytic subunit
**SLC3A2**	solute carrier family 3 member 2; anchor protein
**NCOA4**	nuclear receptor coactivator 4
**MT**	metallothionein
**xCT**	cystine/glutamate antiporter
**CRNDE**	colorectal neoplasia differentially expressed
**CISD2**	CDGSH iron sulfur domain 2
**TACE**	transcatheter arterial chemoembolization
