# Supplementary material for: A comprehensive review of the relationship between autophagy and sorafenib-resistance in hepatocellular carcinoma: ferroptosis is noteworthy
Source: Front Cell Dev Biol. 2023 Apr 27;11:1156383. doi: 10.3389/fcell.2023.1156383 (PMC10172583; doi:10.3389/fcell.2023.1156383)
Supplement: Supplementary file 1 [file Table1.DOCX]

| **Table1．Sorafenib induces autophagy in HCC** | | | | |
| --- | --- | --- | --- | --- |
| **Influence factor** | **Cell line** | **Signal pathway** | **Result** | **Ref** |
| mTOR Pathway | Huh7, HLF, PLC/PRF/5 | PI3K/Akt pathway and mTOR pathway | Sorafenib can not only induce autophagosome formation, but also activate autophagy flux | (28) |
|  | HepG2 | HIF-1/mTOR related signal pathway | Sorafenib can inhibit the proliferation of hepatoma cells and induce autophagy and apoptosis of hepatoma cells through HIF-1/mTOR related signal pathway | (32) |
| Endoplasmic Reticulum Stress | **﻿**MHCC97-L, PLC/PRF/5, HepG2 | Sorafenib induced ER stress and up regulated IRE1 signal | Sorafenib induces apoptosis and autophagy of human hepatoma cells by causing endoplasmic reticulum (ER) stress, which is independent of MEK1/2-ERK1/2 pathway. | (29) |
| Beclin-1Protein | PLC5, Sk-Hep1, HepG2, Hep3B | SHP-1-STAT3-Mcl-1-Beclin1 pathway | Sorafenib and its derivatives induce the inhibition of ml-1 through SHP-1/STAT3 related pathway and release Beclin1 to promote the formation of autophagosome | (31) |
| miR-423-5p | Huh7, HepG2 | **/** | Sorafenib can induce autophagy by affecting mir-423-5p in the treatment of hepatocellular carcinoma | (37) |
| Sphingosine-1-phosphate (S1P) | HepG2 SK-HEP1, Hep 3b2.1-7 | Cell proliferation pathways (such as MAP/ERK pathway) | Sorafenib has some effects on the imbalance of sphingolipid metabolism, which will affect the occurrence of autophagy | (35) |
